# Persons with rheumatoid arthritis have higher barriers to physical activity than controls: a cross-sectional study using the Facilitators and Barriers to Physical Activity Questionnaire (FasBarPAQ)

**DOI:** 10.1007/s00296-022-05252-8

**Published:** 2022-12-07

**Authors:** Vibeke Videm, Ingrid Sæther Houge, Mari Hoff

**Affiliations:** 1 Department of Clinical and Molecular Medicine, NTNU—Norwegian University of Science and Technology, St. Olavs University Hospital, Lab Center 3 East, NO-7006 Trondheim, Norway; 2 Department of Immunology and Transfusion Medicine, St. Olavs University Hospital, Trondheim, Norway; 3 Department of Neuromedicine and Movement Science, NTNU—Norwegian University of Science and Technology, Trondheim, Norway; 4 Department of Rheumatology, St. Olavs University Hospital, Trondheim, Norway

**Keywords:** Rheumatoid arthritis, Physical activity, Patient outcome assessment, Motivation, Questionnaire

## Abstract

Facilitators and barriers to performing physical activity (PA) may vary among persons with rheumatoid arthritis (RA) as well as between RA patients and healthy individuals. Primary objective: To investigate associations of presence of RA and levels of stress and depression with scores for facilitators and barriers to PA, using a new questionnaire (FasBarPAQ). Secondary objectives: investigate inter-individual score differences in persons with RA, and associations with RA disease-specific variables. Persons with RA from two outpatient clinics (*n* = 203) and blood donor controls (*n* = 293) filled in the new 14-item FasBarPAQ questionnaire, the Hospital Anxiety and Depression Scale depression scale (HADS-D), Cohen’s perceived stress scale, and questions regarding PA. Clinical data, and self-reported disease activity and physical function were collected for the persons with RA. Data were analyzed using linear and logistic regression. RA was associated with lower Facilitators scores (coefficient = − 1.30, *p* = 0.015), higher Barriers scores (coefficient = 2.36, *p* < 0.001) and lower Total Facilitators-Barriers scores (coefficient = − 3.67, *p* < 0.001). HADS-D ≥ 8 was associated with lower Total scores (coefficient = − 3.32, *p* = 0.022), and the two higher stress score tertiles were associated with higher Barriers and lower Total scores (*p* = 0.023 to *p* < 0.001). Persons with RA reported greatly varying facilitators and barriers profiles. Seropositivity and higher patient global assessment were associated with higher Barriers scores (coefficients = 1.79, *p* = 0.011; 0.60, *p* < 0.001) and lower Total scores (coefficients = − 3.60, *p* = 0.003; − 0.98, *p* < 0.001). Persons with RA had higher barriers and lower facilitators for PA, with varying individual profiles. The new FasBarPAQ questionnaire may be a useful screening tool for healthcare providers treating persons with RA.

**Supplementary Information:**

The online version contains supplementary material available at 10.1007/s00296-022-05252-8.

## Introduction

Unfavorable lifestyle factors increase the risk of many health-related outcomes, including diabetes, cardiovascular disease (CVD), several forms of cancer, and mental disorders like depression [1–3]. Low physical activity (PA) has emerged as an important such lifestyle factor, in addition for example to smoking, obesity, poor diets, and unhealthy alcohol consumption [1]. Beneficial effects of sufficient PA include improved weight control, increased cardiorespiratory fitness, lower levels of cardiovascular risk factors, improved mental health and sleep, and reduced mortality rates [4–6].

The current general recommendations for aerobic PA are performance either of moderate-intensity activity ≥ 150 min a week or of vigorous-intensity activity ≥ 75 min a week, or a combination of the two, in addition to reduction of sedentary behavior [5, 7]. Only a low percentage of the population in most countries fulfills these recommendations, despite the well-known benefits [8]. In patients with rheumatic diseases, PA performance is even lower. This has been shown for persons with rheumatoid arthritis (RA) [9], a chronic inflammatory condition characterized by joint and general inflammation, as well as comorbidities from the cardiovascular system and lungs, pain, disability, fatigue, and reduced quality of life [10]. The current PA recommendations for persons with RA are similar to the general guidelines [11]. However, persons with RA have significantly lower cardiorespiratory fitness than the rest of the population [9].

Many factors may act as facilitators or barriers to performance of PA, including demographic factors like age and sex, physical factors and comorbidity, and social factors like family and peer support, economy, and access to suitable venues [12–14]. Psychological aspects also play an important role, including exercise self-efficacy, i.e., the extent to which one feels capable of performing PA, motivation, and the levels of perceived stress and depression [12, 15, 16].

One would expect that the relative importance of various facilitators and barriers to PA differs between persons with RA and healthy populations, which could help explain why their level of performed PA is so low. Furthermore, when performing interventions aimed at increasing aerobic PA to recommended levels, assessment of a person’s facilitators and barriers to PA is important as individual differences preclude a “one-size-fits-all” approach. Therefore, simple tools to assess facilitators and barriers to PA on a personal level are needed.

Our main hypothesis was that by developing a simple screening instrument, we would be able to show general differences between persons with RA and healthy controls with respect to common facilitators and barriers for PA, and that perceived stress and depression were associated with the assessment scores. We also hypothesized that there would be substantial inter-individual differences among persons with RA, and that RA-specific variables would influence the scores. Finally, we hypothesized that the scores would be associated with fulfillment or not of the PA recommendations.

The primary aim of the present study was therefore to investigate associations of presence or absence of RA and interactions with levels of stress and depression, with scores for facilitators and barriers to PA. Secondary aims were to investigate inter-individual differences in scores for facilitators and barriers to PA in persons with RA, whether RA disease-specific variables were associated with the scores, and whether the scores were associated with fulfillment of PA recommendations in RA patients and controls.

## Methods

The present sub-study is part of a larger ongoing cross-sectional study of patient-related outcome measures and PA in patients with inflammatory arthritis, Fyskond2. Data from a subgroup of Fyskond2 participants have been used in a previous publication [17].

### Patients and controls

Participants with complete data for the Facilitators and Barriers to PA Questionnaire (FasBarPAQ, further described below) by 31.12.2021 were included in this sub-study. Blood donors were included to represent healthy controls as they are easily accessible and have no major comorbidities. They were approached during a routine visit to the Blood Bank at St. Olavs University Hospital in Trondheim, Norway in 2019, and provided anonymous data for the study. The only inclusion criterion for the blood donors was willingness to participate, and there were no exclusion criteria. The number of controls was pre-defined to *n* = 300. Persons with RA fulfilling the EULAR/ACR 2010 criteria [18] from the Rheumatology clinics at Levanger Hospital or St. Olavs University Hospital received written information about the study. Participants were thereafter recruited from RA patients with scheduled outpatient appointments (2019–2021), or from a randomly selected list of RA patients following the patient-centered follow-up program for persons with inflammatory arthritis at St. Olavs University Hospital (2021). The latter patients contact the rheumatology outpatient clinic when their disease is active and are otherwise followed up by their general practitioner. Both patient groups were included to ensure a wide range of characteristics and disease activity among the persons with RA and thereby better generalizability of the results. The inclusion criteria for the patients were an ascertained RA diagnosis and being willing to participate, and there were no exclusion criteria. Due to the covid-19 pandemic, some participants were approached by mail and submitted their questionnaires in a return envelope.

### Main outcome variable

The main outcome variables were the three scores from the FasBarPAQ, i.e. the Facilitators score, the Barriers score, and the Total Facilitators minus Barriers score. In detail, facilitators and barriers to PA in persons with RA were first investigated in a pilot part of a previous study on cardiopulmonary fitness [19], where participants were invited to give brief free-text input. Based on common themes and an extensive literature search, the present 14 items were formulated and included in the FasBarPAQ (Table 1). Responses were given as Likert scales ranging from 0 (totally disagree) to 4 (totally agree). In another pilot study, wording and completeness of replies to the FasBarPAQ were anonymously tested in *n* = 308 students from the Faculty of Medicine and Health Sciences and Faculty of Information Technology and Electrical Engineering at NTNU—Norwegian University of Science and Technology. No problematic issues were identified, but preliminary exploratory factor analysis indicated that several items loaded on more than one factor. The FasBarPAQ was therefore included in Fyskond2 and scored as follows: reponses to positively worded items (*n* = 7) were added to a Facilitators score (potential range: 0, 28), responses to negatively worded items (*n* = 7) were added to a Barriers score (potential range: 0, 28), and a total score was calculated as the Facilitators score minus the Barriers score (potential range: − 28, 28).

**Table 1:** Item wording and observed scores^a^

Item number^b^	RA patients *n* = 203	Blood donors *n* = 293	*p* value^c^ RA vs. blood donors
1: Support from friends and family means a lot for my physical activity	2 (1, 3)	2 (1, 2)	*p* = 0.19
	*1.8 (1.4)*	*1.7 (1.2)*	
2: Physical activity gives me a sense of well-being and increased energy	4 (3, 4)	4 (3, 4)	*p* < 0.001
	*3.2 (1.0)*	*3.5 (0.7)*	
3: I don’t have enough time for physical activity	0 (0, 2)	1 (0, 2)	*p* < 0.01
	*0.9 (1.1)*	*1.1 (1.1)*	
4: I experience less pain when I’m physically active	2 (2, 4)	3 (2, 4)	*p* = 0.25
	*2.6 (1.3)*	*2.7 (1.2)*	
5: I’m concerned that physical activity will worsen my disease-related ailments	0 (0, 1)	0 (0, 0)	*p* < 0.001
	*0.6 (1.0)*	*0.2 (0.6)*	
6: I think physical activity is fun	3 (2, 4)	3 (2, 4)	*p = *0.041
	*2.8 (1.2)*	*3.1 (0.9)*	
7: My body limits how physically active I can be	2 (1, 4)	1 (0, 2)	*p* < 0.001
	*2.3 (1.3)*	*0.9 (1.1)*	
8: Participating in physical activity contributes to my having nice social interactions	2 (2, 4)	2 (2, 3)	*p* = 0.14
	*2.6 (1.3)*	*2.4 (1.2)*	
9: I’m too tired and worn out to be physically active	1 (0, 2)	0 (0, 1)	*p* < 0.001
	*1.2 (1.1)*	*0.6 (0.8)*	
10: I become less stressed and/or sleep better if I’m physically active	3 (2, 4)	4 (3, 4)	*p* < 0.001
	*2.9 (1.1)*	*3.3 (0.9)*	
11: I need clear advice on how to train to be physically active	1 (0, 2)	1 (0, 2)	*p* < 0.01
	*1.2 (1.3)*	*0.9 (1.1)*	
12: Few available activities and/or long travel time limits how physically active I can be	0 (0, 0)	0 (0, 0)	*p* = 0.72
	*0.5 (1.1)*	*0.4 (0.8)*	
13: Being physically active gives me a feeling of independence and/or control	2 (2, 3)	3 (2, 3)	*p* = 0.047
	*2.4 (1.2)*	*2.6 (1.1)*	
14: Physical activity means a lot for preventing further health problems	3 (2, 4)	3 (2, 4)	*p* < 0.01
	*3.1 (1.0)*	*2.8 (1.2)*	

^a^Upper line gives median (25th and 75th percentile), lower line (italics) gives mean (SD)

^b^Facilitators: items 2, 4, 6, 8, 10, 13, 14; barriers: items 1, 3, 5, 7, 9, 11, 12

^c^ Mann–Whitney *U* test

The wording of the FasBarPAQ was carefully chosen not to mention arthritis specifically, because the literature supports that many facilitators and barriers to PA are widely relevant. We therefore considered that the questionnaire could be useful in general populations and other patient groups, for example individuals with other musculoskeletal diseases or conditions involving pain. The complete questionnaire is included in Online Resource 1 (Norwegian and English versions) and may be used freely when referenced to the present publication.

### Study factors and other variables

For all participants, the Hospital Anxiety and Depression Scale (HADS) depression score [20] was used to assess depressive symptoms during the past week. Based on 7 items, it ranges from 0–21 with higher scores implying more depressive symptoms. Scores ≥ 8 define possible clinical cases with a sensitivity and specificity ~ 0.70–0.90 [21]. Cohen's scale for perceived stress was used to assess perceived stress during the last month [22]. Based on 10 questions, it ranges from 0–40 with higher scores implying more perceived stress. To evaluate whether participants fulfilled the recommendations for PA from the American College of Cardiology and the American Heart Association (ACC/AHA) [7], information about frequency, duration, and intensity of the participants' habitual PA was collected.

For participants with RA, self-reported physical function during the past week was measured using the modified Stanford Health Assessment Questionnaire (mHAQ) [23]. Disease activity was quantified using the patient global assessment (PGA) on a 0–100 mm visual analog scale in response to the phrases, “Please consider the activity of your rheumatic disease in the past week. When considering all the symptoms, how do you think your state is?” Because some of the included patients had not recently visited a rheumatologist and some participated by mail, joint scores and measurements of C-reactive protein or erythrocyte sedimentation rate were not available. Thus, the disease activity score-28 (DAS28) could not be calculated. Patients rated their present level of joint pain on a Likert scale from 0 to 10, where 0 indicated “no pain” and 10 indicated “very intense pain.” Hospital records were reviewed for information regarding the diagnosis, anti-rheumatic medication, comorbidities (yes/no variable including history of hypertension, angina, myocardial infarction, arrythmia, stroke, chronic obstructive or chronic restrictive pulmonary disease and/or cancer), and seropositivity status (positive test for rheumatoid factor and/or anti-citrullinated peptide antibody).

### Procedures

Fyskond2 is performed in accordance with the Helsinki declaration. It was approved by the Regional Committee for Medical and Health Research Ethics (#23420). Participants give informed consent before inclusion.

### Statistical analysis

Most continuous variables were not normally distributed in histograms and are therefore given as median (25th and 75th percentile) and were compared using the Mann–Whitney *U* test. To get more granularity of the scores and render other data comparable to the literature, tables also include mean (SD). Categorical data are given as number (%) and were compared using the X^2^ test. Linear correlation between the Facilitators and Barriers scores was evaluated with Pearson's correlation coefficient. Due to low levels of missingness, complete data were analyzed (details below).

Further analysis was performed using linear regression modeling. The dependent variables were each of the Facilitators, Barriers, and total Facilitators-Barriers scores. For the primary aim of investigating whether RA, depression, and stress were associated with the scores, the explanatory variables in each model were presence of RA (yes/no), HADS depression score ≥ 8 (yes/no), and Cohen’s perceived stress score (categorized as tertiles: 0–8, 9–14, 15–40), with adjustments for sex (0 = female, 1 = male) and age (categorized in tertiles: ≤ 45, 46–60, > 60 years). Tertiles were used to achieve good model fit. Interactions between RA and depression or stress scores were tested with inclusion of a product term (RA × depression score or RA × stress score).

Interindividual score profiles in persons with RA were assessed using the spread in responses and illustrated using radar plots of individuals with identical total scores. For the secondary aim of investigating associations between RA-specific variables and each of the scores in persons with RA, the prespecified explanatory variables were duration of RA, age at RA diagnosis, seropositivity (yes/no), PGA, and sex, with adjustments for age tertile, comorbidity (yes/no), and use of conventional and/or biological disease-modifying anti-rheumatic drugs (yes/no). In a sensitivity analysis, mHAQ and the joint pain scores were also included in the models.

For the secondary aim of assessing whether the Facilitators and Barriers scores were associated with fulfillment or not of the ACC/AHH recommendations for PA, logistic regression was used. The dependent variable was fulfillment of the recommendations (yes/no). The exploratory variables were presence of RA (yes/no), values of the two scores, and sex, with adjustment for age tertile.

Assumptions for the linear regression models were evaluated using residual plots. Linearity of logits in the logistic regression models was assessed using plots. Data were analyzed using Stata (v.16.0, Statacorp, College Station, TX, USA). P-values < 0.05 were considered significant.

#### Results

Data from the FasBarPAQ were available for *n* = 496 participants, including *n* = 203 persons with RA and *n* = 293 blood donors. For further analysis comparing the two groups, *n* = 484, because 9 persons with RA (4.4%) and 3 blood donors (1.0%) were excluded due to other missing variables (blood donors: depression score and stress score *n* = 3; persons with RA: depression score: *n* = 4, stress score: *n* = 7). For analysis in RA patients only, *n* = 194 (missing data: PGA *n* = 5, seropositivity: *n* = 2, duration of RA: *n* = 2).

Participant characteristics are given in Table 2. The persons with RA were older than the blood donors, more were women or ever smokers, and they had higher depression and stress scores. Median RA duration was 11 years, ~ 80% of the RA patients were seropositive, and many had comorbidities. The majority were treated with disease-modifying anti-rheumatic drugs (DMARD), median PGA was 28 mm, and median HAQ was 0.25. To account for the sex and age differences between the persons with RA and the controls, the multivariable analyses reported below were adjusted for these variables. Adjustment for smoking was not performed because smoking has complex associations with several other variables in the study. For example, smoking is a risk factor for RA, and may be a mediator between depression and reduced performance of PA.

**Table 2:** Participant characteristics^a^

Variable	RA patients (*n* = 203)	Blood donors (*n* = 293)	*p* value^b^
Age, years	62 (53,70)	45 (34, 54)	< 0.001
	*60 (13)*	*44 (14)*	
Age tertile, *n* (%)			
1: ≤ 45 years	28 (13.8%)	149 (50.9%)	< 0.001
2: 46–60 years	62 (30.5%)	104 (35.5%)	
3: > 60 years	113 (55.7%)	40 (13.6%)	
Women, *n* (%)	138 (68.0%)	159 (54.3%)	0.002
Body mass index, kg/m^2^	26.0 (23.3, 28.9)	25.8 (23.3, 28.9)	0.90
	*26.5 (4.7)*	*26.3 (3.9)*	
Ever smoker	127 (62.6%)	102 (34.8%)	< 0.001
Duration of RA, years	11 (5, 19)	–	
	*13 (11)*		
Age at RA diagnosis, years	47 (36, 58)	–	
	*47 (15)*		
Seropositive (rheumatoid factor and/or anti-citrullinated peptide antibody positive), *n* (%)	162 (79.8%)	–	
Comorbidity, *n *(%)			
Hypertension	59 (29.1%)	–	
Cardiovascular disease^c^	80 (39.4%)		
Diabetes	10 (4.9%)		
Respiratory disease^d^	35 (17.2%)		
Cancer	20 (9.9%)		
Current medication, *n* (%)			
Conventional DMARDs	168 (82.8%)	–	
Biological DMARDs	103 (50.7%)		
Patient global assessment (mm)	28 (13, 49)	–	
	*32 (23)*		
Modified health assessment questionnaire	0.25 (0, 0.63)	–	
Joint pain score (0–10; 0 = no pain, 10 = very intense pain)	*0.41 (0.43)*		
	2 (1, 5)		
	*3.2 (2.6)*		
HADS depression score	3 (1, 5)	1 (0, 3)	< 0.001
	*4 (3)*	*2 (2)*	
HADS depression score ≥ 8, *n* (%)	23 (11.6%)	12 (4.1%)	0.002
Cohen’s perceived stress score	14 (8, 18)	10 (7, 14)	< 0.001
	*14 (6)*	*10 (6)*	
Cohen’s perceived stress score tertile, *n* (%)			
1: 0–8	50 (25.6%)	113 (38.6%)	< 0.001
2: 9–14	58 (28.6%)	120 (41.0%)	
3: 15–40	88 (43.7%)	57 (19.5%)	
Facilitators score (possible range: 0, 28)	21 (16, 24)	21 (18, 24)	0.31
	*19.7 (5.7)*	*20.5 (4.4)*	
Barriers score (possible range: 0, 28)	8 (5, 11)	5 (3, 8)	< 0.001
	*8.5 (4.2)*	*5.9 (3.7)*	
Total Facilitators-Barriers score (possible range: − 28, 28)	12 (5, 17)	16 (10, 19)	< 0.001
	*11.2 (7.6)*	*14.7 (6.3)*	
Fulfills ACC/AHH recommendations for physical activity	57 (28.1%)	102 (34.8%)	0.11

*ACC/AHA* American College of Cardiology/American Heart Association, *DMARD* disease-modifying anti-rheumatic drug, *HADS* Hospital Anxiety and Depression Scale, *RA* rheumatoid arthritis

^a^Continuous variables: upper line gives median (25th and 75th percentile), lower line (italics) gives mean (SD). Categorical variables: number (%). Missing data: RA patients: smoking *n* = 2, Cohen’s perceived stress score *n* = 7; controls: smoking *n* = 2, Cohen’s perceived stress score *n* = 3

^b^Mann-Whitney *U* test or X^2^ test

^c^chronic obstructive or chronic restrictive pulmonary disease

^d^angina, myocardial infarction, arrythmia, stroke

##### Primary aim

**Fig. 1:**
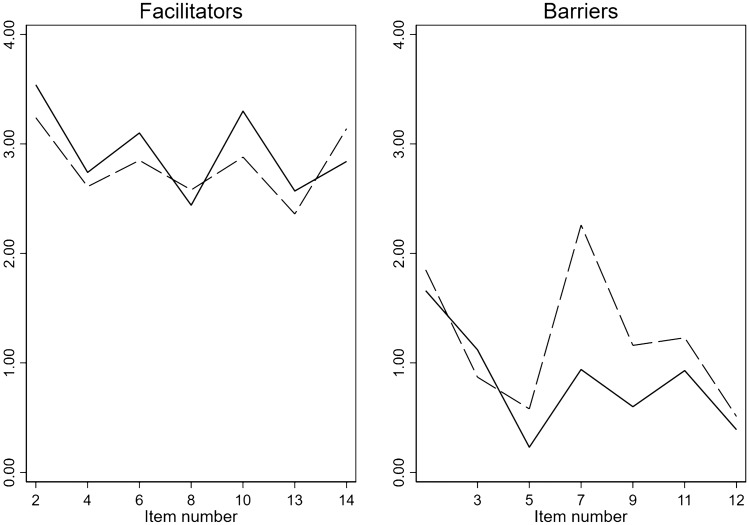
Unadjusted facilitators and barriers item scores. Mean scores in RA patients (*n* = 203, solid line) and blood donors (*n* = 293, dashed line)

Observed scores for the individual Facilitators and Barriers to PA items are shown in Table 1 and Fig. 1. The facilitators related to well-being/energy (item # 2) and less stress/better sleep (item # 10) from PA were lower in persons with RA, but they scored higher on performing PA to prevent further health problems (item # 14). The barriers regarding body limits (item # 7), being too tired/worn out for PA (item # 9) and need for clear advice for PA (item # 11) were higher in persons with RA compared to blood donors. Even if several item differences were not very large, the general response patterns resulted in significantly higher unadjusted Barriers scores (*p* < 0.001) and lower unadjusted total Facilitators-Barriers scores (*p* < 0.001, Table 2) in the persons with RA. The Facilitators and Barriers scores were weakly linearly correlated (*R* = − 0.15, *p* < 0.001).

**Fig. 2:**
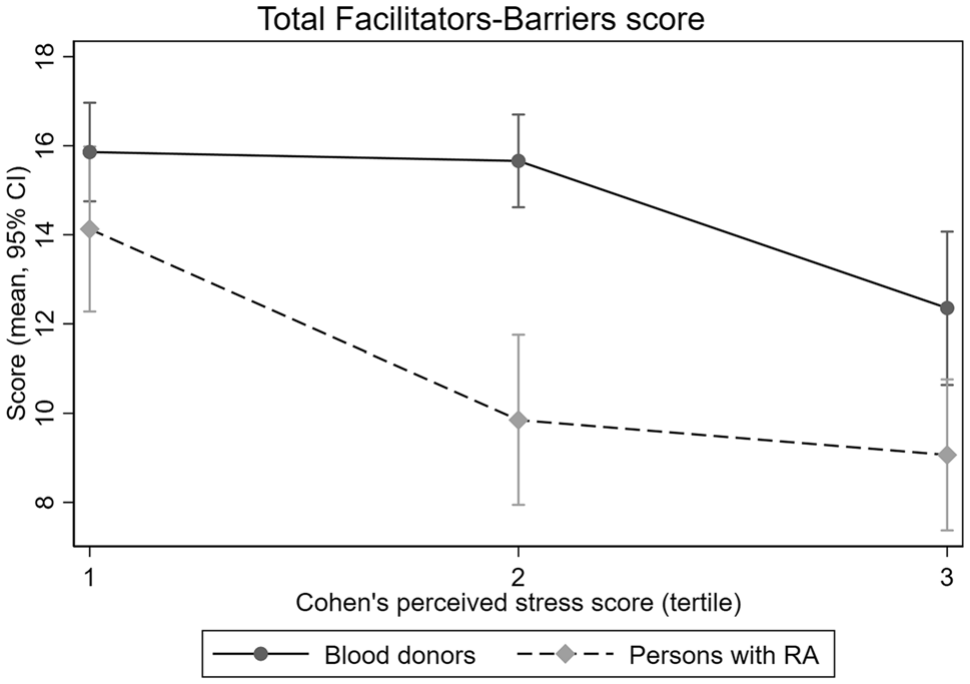
Total Facilitators-Barriers score and tertile of perceived stress. Mean scores from regression model in RA patients (*n* = 194,) and blood donors (*n* = 290), showing a significant interaction effect (*p* = 0.023). The model also included the depression score from the Hospital Anxiety and Depression Scale, and adjustments for age and sex

Table 3 gives results from the adjusted linear regression analyses comparing persons with RA and blood donors. In these multivariable models with adjustment for age and sex, having RA was associated with lower Facilitators scores (*p* = 0.015), higher Barriers scores (*p* < 0.001), and lower total Facilitators-Barriers scores (*p* < 0.001). Men scored lower on Facilitators (*p* = 0.001), leading to lower total scores (*p* = 0.008), whereas older participants had higher total scores (*p* < 0.001). Depression scores ≥ 8 and higher perceived stress were associated with significantly lower total Facilitator-Barriers scores (Table 3). Higher perceived stress was also associated with higher Barriers scores. When adding interactions between RA and depression or stress scores to these models, a significant interaction was found for the association of RA and perceived stress with the total Facilitators-Barriers score (*p* = 0.023): total scores in persons with RA dropped significantly from the first to second stress score tertile and then remained constant, whereas only blood donors in the highest tertile had a similar drop in total scores (Fig. 2).

**Table 3:** Linear regression models

Variable	Facilitators score		Barriers score		Total Facilitators-Barriers score	
	Coefficient (95% CI)	*P* value	Coefficient (95% CI)	*P* value	Coefficient (95% CI)	*P* value
**Primary analysis: models for all participants**^a^						
RA	− 1.30 (− 2.34, − 0.26)	0.015	2.36 (1.55, 3.18)	< 0.001	− 3.67 (− 5.09, − 2.25)	< 0.001
Male sex	− 1.60 (− 2.52, − 0.70)	0.001	0.01 (− 0.65, 0.66)	0.98	− 1.60 (− 2.79, − 0.41)	0.008
Age tertile						
1	Reference		Reference		Reference	
2	1.37 (0.36, 2.38)	0.008	− 1.20 (− 2.01, − 0.40)	0.003	2.58 (1.18, 3.97)	< 0.001
3	1.58 (0.34, 2.81)	0.012	1.51 (− 2.48, − 0.54)	0.002	3.08 (1.37, 4.80)	< 0.001
HADS depression score above median	− 2.01 (− 4.18, 0.17)	0.070	1.31 (− 0.33, 2.95)	0.12	− 3.32 (− 6.14, − 0.49)	0.022
Cohen’s perceived stress score tertile						
1	Reference		Reference		Reference	
2	− 0.70 (− 1.71, 0.30)	0.17	0.79 (0.08, 1.49)	0.029	− 1.49 (− 2.81, − 0.17)	0.027
3	− 0.60 (− 1.84, 0.63)	0.34	3.22 (2.30, 4.14)	< 0.001	− 3.82 (− 5.44, − 2.20)	< 0.001
**Secondary analysis: models for persons with RA only**^b^						
Duration of RA (per year)	0.06 (− 0.09, 0.20)	0.44	− 0.05 (− 0.17, 0.06)	0.34	0.11 (− 0.08, 0.31)	0.26
Age at RA diagnosis (per year)	0.04 (− 0.11, 0.19)	0.63	− 0.04 (− 0.14, 0.06)	0.38	0.08 (− 0.11, 0.28)	0.41
Seropositive^c^	− 1.81 (− 3.73, 0.11)	0.065	1.79 (0.41, 3.18)	0.011	− 3.60 (− 5.96, − 1.24)	0.003
Patient global assessment (per 10 mm)	− 0.37 (− 0.75, 0.00)	0.052	0.68 (0.47, 0.90)	< 0.001	− 0.98 (− 1.42, − 0.53)	< 0.001

*CI* confidence interval, *HADS* hospital anxiety and depression scale, *RA* rheumatoid arthritis

^a^Multivariable models for each of the three scores, including patients with RA (*n* = 194) and controls (*n* = 290)

^b^Multivariable models for each of the three scores, including patients with RA only (*n* = 194). These models were adjusted for age in tertiles, comorbidity yes/no (history of hypertension, angina, myocardial infarction, arrythmia, stroke, chronic obstructive or chronic restrictive pulmonary disease, diabetes, and/or cancer), and use of conventional and/or biological disease-modifying anti-rheumatic drugs

^c^Positive for rheumatoid factor and/or cyclic citrullinated peptide antibody

##### Secondary aims

**Fig. 3:**
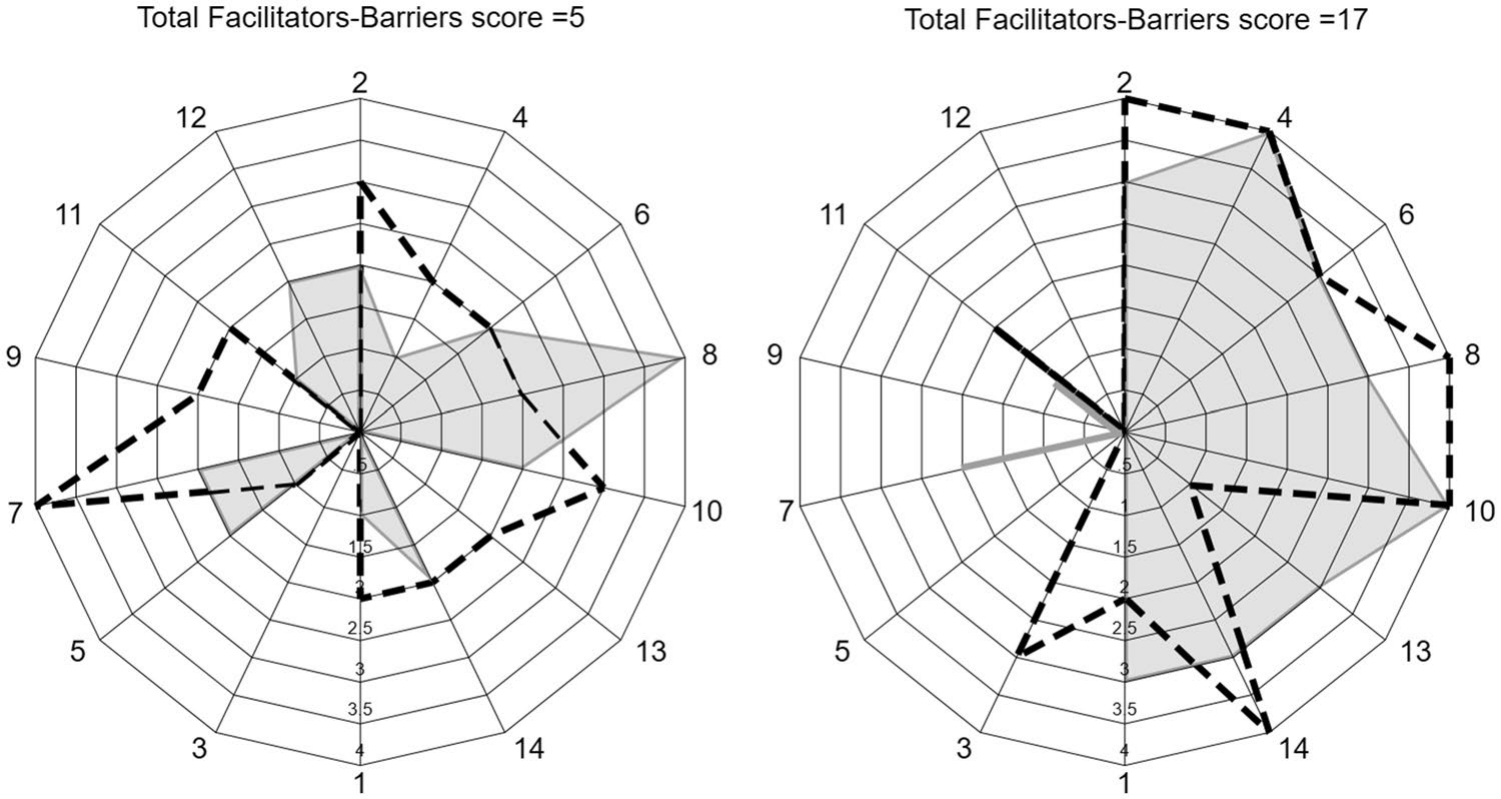
Examples of item profiles for 4 persons with rheumatoid arthritis (RA). Each spike in the radar plots corresponds to an item in the Facilitator and Barriers to Physical Activity Questionnaire (FasBarPAQ). Items 2, 4, 6, 8, 10, 13, and 14 (right-hand side of plots) represent facilitators. Items 1, 3, 5, 7, 9, 11, and 12 (left-hand side of plots) represent barriers. Left-hand plot: scores from 2 individuals with total score = 5 (25th percentile in the study). Marked by shaded area: woman 71 years, seropositive RA, duration 15 years. Marked by dashed line: woman 64 years, seropositive RA, duration > 40 years. Right-hand plot: scores from 2 individuals with total score = 17 (75th percentile in the study). Marked by shaded area: woman 59 years, seronegative RA, duration 36 years. Marked by dashed line: woman 51 years, seropositive RA, duration 15 years

Figure 3 shows examples of individual item scores for 4 persons with RA, including 2 persons with total Facilitators-Barriers scores of 5 (25th percentile) and 2 persons with total scores of 17 (75th percentile). The figure demonstrates large individual differences in responses to each item comprising the total score, indicating that not only the total score, but the item profile for each person is of importance. A radar plot showing mean values of all items from the blood donors is included in Online Resource 2, and may be used for comparison with scores for individual persons with RA to identify person-specific themes for intervention.

Results regarding the secondary aim of investigating associations of RA-specific variables and the Facilitators, Barriers and total Facilitators-Barriers scores are given in Table 3. Seropositivity and higher PGA were associated with higher Barriers scores (*p* = 0.011 and *p* < 0.001, respectively) and lower total scores (*p* = 0.003 and *p* < 0.001, respectively). Duration of RA and age at RA diagnosis showed no significant associations (*p* ≥ 0.26). The sensitivity analysis with inclusion of mHAQ scores to the models showed that this factor was not significant (*p* ≥ 0.06). Inclusion of the pain score induced collinearity and distorted the models due to close correlation with the PGA (*R* = 0.80, *p* < 0.001).

Approximately 1/3 of the participants fulfilled the ACC/AHA recommendations for aerobic PA, and there was no significant intergroup difference (*p* = 0.11, Table 2). The multivariable logistic regression model also including sex and age tertiles showed that higher Facilitators scores (Odds ratio 1.19 per point, 95% CI 1.13,1.25, *p* < 0.001) and lower Barriers scores (Odds ratio 0.85 per point, 95% CI 0.79,0.90, *p* < 0.001) were independently associated with fulfillment of the ACC/AHA recommendations. There was no association with RA (*p* = 0.23), but males more often fulfilled the recommendations than women (Odds ratio 2.44, 95% CI 1.56, 3.81, *p* < 0.001). The area under the receiver operating characteristic curve was 0.77 (95% CI 0.73, 0.81), which indicates good discrimination.

Further information regarding validation of the FasBarPAQ is given in Online Resource 3.

## Discussion

The present study showed that presence of RA was associated with lower Facilitators to PA scores, higher Barriers scores, and lower total Facilitator-Barriers scores compared to a healthy control group in models adjusted for age and sex. Higher perceived stress and depression scores were associated with lower total Facilitator-Barriers scores. Lower levels of stress were associated with reduced total scores in RA patients compared to controls. Furthermore, seropositivity and higher PGA were associated with higher Barriers scores and lower total scores. Duration of RA and age at diagnosis were not associated with the scores. The Facilitators and Barriers scores were only weakly correlated. Higher Facilitators scores and lower Barriers scores were associated with fulfillment of the recommendations for aerobic PA both in persons with RA and controls.

### Facilitators and barriers to PA in RA

The findings from the study support our hypothesis that increased barriers and weaker facilitators may be an important reason for less performance of PA in persons with RA. The FasBarPAQ provides a simple and quick way for healthcare providers to screen individual patients. The total score helps identify persons with high barriers and few facilitators, and the item scores help identify areas for intervention independent of total scores. For optimal usefulness, relevant responses should be further explored in a conversation with the patient.

The study showed that facilitators and barriers should be considered as different issues because the two scores were only weakly correlated. It is important to address barriers, but also to help the person plan activities based on facilitators, which may increase the likelihood of adherence. People have individual preferences with respect to PA [24, 25]. We may hypothesize that the questionnaire can offer a structured way to access input for personalized training programs. We may also speculate that the questionnaire may prove to be helpful when evaluating effects of interventions like motivational interviews, patient education programs, and implementation of self-management strategies, which are essential to patient-centered treatment of inflammatory arthritis [26, 27]. Performance of the FasBarPAQ in such settings needs further evaluation in future studies.

The negative influence of stress on PA is in accordance with previous reports [28]. Our study showed that the total Facilitators-Barriers score dropped at lower levels of perceived stress in persons with RA than controls. This is an important finding because several suggested stress management methods are relevant also for RA patients, including improved sleep hygiene, strengthening of social connections, and mind-body practices [29]. Furthermore, exercise in itself is a tool for stress management [28, 29], underscoring the need for a multifaceted lifestyle approach as part of RA care.

The relationship between depression and PA is complicated and may also be bi-directional both in persons with RA [30] and other populations [16, 31]. Many persons with RA have depressive symptoms [10, 30], which may also be part of the explanation for their low levels of PA. Depression is amenable to treatment but may be underdiagnosed in RA [30]. The levels of depression as indicated by HADS depression scores ≥ 8 in the present study were low both for persons with RA and controls [30, 32], which may be due to recruitment bias and blood donor selection criteria.

Higher RA disease activity is associated with more pain and physical limitation. This may explain the association of seropositive RA with higher barriers to PA, because seropositive disease is associated with increased joint damage [10]. The PGA is a subjective summary measure where patients may include aspects like disease activity, function, symptoms, and psychological factors [33]. These may all impact barriers and facilitators to PA, explaining the observed associations with the scores. It seems like this personal evaluation was more important than objective measures like age at diagnosis and RA duration, confirming the high relevance of patient-related outcome measures for attitudes towards PA.

### Comparison with other questionnaires

Other relevant questionnaires for facilitators and barriers to PA have been published, including a general 43-item questionnaire in 1987 [34], a 38-item questionnaire for persons with osteoarthritis in 2017 [35], and a 10-item questionnaire for persons with inflammatory arthritis (IFAB) in 2020 [36]. Even though many of the items in the 43-item questionnaire are relevant to persons with RA [34], we considered it as too extensive to be practical in our everyday practice. Furthermore, the results are given as 9 sub-scores whereas we found that assessment of single items in addition to calculation of a total score would be most clinically useful. The questionnaire for OA covers 6 domains [35] and would need validation for other diagnoses. It was also considered as too extensive.

Our study was ongoing before the IFAB questionnaire was published [36]. IFAB was validated in a small patient group comprising 26 persons with RA, 24 persons with axial spondylarthritis, and 13 individuals with psoriasis arthritis. Thus, our study was much larger and included a non-IA group for comparison. On the other hand, the IFAB performed well in a later larger study [37]. The differences between the issues covered in our questionnaire and IFAB are relatively small. The IFAB focuses on experiences during the last month whereas the FasBarPAQ is more open. Only a future direct comparison can evaluate whether they are exchangeable.

### Strengths and limitations

The inclusion of large study groups of persons with validated RA and healthy controls is a strength. The recruitment procedure permitted inclusion of patients with widely varying levels of RA activity, even if we missed more objective variables such as swollen joint counts and the DAS28 that would have been obtainable if we only recruited patients visiting a rheumatology clinic. The FasBarPAQ may be useful in populations with different characteristics because it is not dependent on specific score cutoff levels or profiles, and covers many generally relevant facilitators and barriers to PA for persons with and without RA. Our control values may not fit as references in other countries because of cultural differences regarding facilitators and barriers to PA, so local reference data may be needed. However, the FasBarPAQ can be used in individual persons without the need for reference data. Before use in patient groups with other diagnoses, validation would be necessary.

Self-reported data including self-reported PA may have low accuracy [38]. A study from the UK showed that as they grow older, middle-aged adults increasingly tend to over-report their level and intensity of PA compared to accelerometer data [39]. This tendency may have biased our findings, which is a limitation. A better test of validity would therefore have been to evaluate the association of the FasBarPAQ results with fulfillment of PA recommendations using an objective test of cardiopulmonary fitness or accelerometer data. Unfortunately, resources for such measurements were not available. Self-reported scores for stress and depression may also have low accuracy, but the employed instruments have been extensively used in previous research.

Blood donors are not representative for the general population because persons with diagnoses or medication use that influence donor or blood recipient safety are excluded. They may still be relevant as controls for persons with inflammatory arthritis because there are few restrictions related to symptoms of everyday musculoskeletal complaints or osteoarthritis, and symptoms that are not associated with serious diagnoses [40]. Furthermore, the statistical models were adjusted for age and sex to account for the differences between the persons with RA and controls.

We cannot exclude that inclusion of more or other items could have influenced the findings and that there may be residual confounding. The association of the scores with fulfillment of aerobic PA recommendations and the differences in scores between the persons with RA and controls underscore the validity and usefulness of the FasBarPAQ, but the questionnaire is not intended for prediction of this endpoint.

## Conclusions

Increased barriers and weaker facilitators may be important reasons for less performance of PA in persons with RA. To help patients achieve their targets for PA, it is important to address their individual barriers and build on their personal facilitators, but also address their levels of perceived stress and depression. The FasBarPAQ provides a simple and quick way for healthcare providers to screen a wide range of facilitators and barriers to PA in individual patients.

## Supplementary Information

Below is the link to the electronic supplementary material.

Supplementary file1 (PDF 130 KB)

Supplementary file2 (PDF 171 KB)

Supplementary file3 (PDF 114 KB)

## Data Availability

No additional data are available.
