# Supplementary material for: Persons with rheumatoid arthritis have higher barriers to physical activity than controls: a cross-sectional study using the Facilitators and Barriers to Physical Activity Questionnaire (FasBarPAQ)
Source: Rheumatol Int. 2022 Dec 7;43(2):303–14. doi: 10.1007/s00296-022-05252-8 (PMC9734883; doi:10.1007/s00296-022-05252-8)
Supplement: Supplementary file 1 — Supplementary file1 (PDF 130 KB) [file 296_2022_5252_MOESM1_ESM.pdf]

Videm V, Houge IS, Hoff M: Persons with rheumatoid arthritis have higher barriers to physical activity than controls – a cross-sectional study using the Facilitators and Barriers to Physical Activity Questionnaire (FasBarPAQ)
Rheumatology International
Corresponding author: Vibeke Videm, Department of Clinical and Molecular Medicine, NTNU – Norwegian University of Science and Technology and Department of Immunology and Transfusion Medicine, St. Olavs University Hospital, Trondheim, Norway. E-mail: vibeke.videm@ntnu.no

Online Resource 1: Facilitators and Barriers to Physical Activity Questionnaire in Norwegian and English

Forhold som gjør det lettere eller vanskeligere for meg å trene eller være fysisk aktiv

Nedenfor finner du en liste over utsagn om forhold som kan gjøre det lettere eller vanskeligere å være fysisk aktiv. Med fysisk aktivitet menes både trening og mosjon. Vennligst angi hvor enig eller uenig du er i hvert utsagn ved å sette kryss i én av rutene på skalaen på hver linje. Det finnes ingen riktige eller gale svar – vi ønsker bare å få en beskrivelse av hva som passer best for deg.

	Helt uenig		Delvis enig		Helt enig
1) Støtte fra familie og venner betyr mye for at jeg skal være fysisk aktiv	☐	☐	☐	☐	☐
2 Fysisk aktivitet gir meg velvære og energi	☐	☐	☐	☐	☐
3) Jeg har for dårlig tid til fysisk aktivitet	☐	☐	☐	☐	☐
4) Jeg får mindre smerter hvis jeg er fysisk aktiv	☐	☐	☐	☐	☐
5) Jeg er urolig for at fysisk aktivitet skal forverre sykdomsplagene mine	☐	☐	☐	☐	☐
6) Jeg synes fysisk aktivitet er gøy	☐	☐	☐	☐	☐
7) Kroppen min setter begrensninger for hvor fysisk aktiv jeg kan være	☐	☐	☐	☐	☐
8) Deltakelse i fysisk aktivitet bidrar til at jeg får hyggelig sosial kontakt	☐	☐	☐	☐	☐
9) Jeg er for trett og sliten til å være fysisk aktiv	☐	☐	☐	☐	☐
10) Jeg blir mindre stresset og / eller sover bedre hvis jeg er fysisk aktiv	☐	☐	☐	☐	☐
11) Jeg trenger klare treningsråd for å kunne være fysisk aktiv	☐	☐	☐	☐	☐

12) Dårlige aktivitetstilbud og / eller lang reisevei begrenser hvor fysisk aktiv jeg kan være	☐	☐	☐	☐	☐
13) Å være fysisk aktiv gir meg en følelse av uavhengighet og / eller kontroll	☐	☐	☐	☐	☐
14) Fysisk aktivitet betyr mye for at jeg ikke skal få større helseplager	☐	☐	☐	☐	☐

Conditions that make it easier or harder for me to work out or be physically active

Below you will find a list of statements regarding issues that may make it easier or harder to be physically active. Physical activity includes both working out and performing exercise. Please mark to what extent you agree or disagree with each statement by placing one X on each line of boxes of the form. There are no right or wrong answers; we only want a description of what fits best for you.

	Totally disagree		Partly agree		Totally agree
1) Support from friends and family means a lot for my physical activity	☐	☐	☐	☐	☐
2) Physical activity gives me a sense of well-being and increased energy.	☐	☐	☐	☐	☐
3) I don't have enough time for physical activity.	☐	☐	☐	☐	☐
4) I experience less pain when I'm physically active . .	☐	☐	☐	☐	☐
5) I'm concerned that physical activity will worsen my disease-related ailments.	☐	☐	☐	☐	☐
6) I think physical activity is fun	☐	☐	☐	☐	☐
7) My body limits how physically active I can be.	☐	☐	☐	☐	☐
8) Participating in physical activity contributes to my having nice social interactions.	☐	☐	☐	☐	☐
9) I'm too tired and worn out to be physically active . .	☐	☐	☐	☐	☐
10) I become less stressed and/or sleep better if I'm physically active.	☐	☐	☐	☐	☐
11) I need clear advice on how to train to be physically active.	☐	☐	☐	☐	☐
12) Few available activities and/or long travel time limits how physically active I can be.	☐	☐	☐	☐	☐
13) Being physically active gives me a feeling of independence and/or control.	☐	☐	☐	☐	☐
14) Physical activity means a lot for preventing further health problems.	☐	☐	☐	☐	☐
