# Supplementary material for: Persons with rheumatoid arthritis have higher barriers to physical activity than controls: a cross-sectional study using the Facilitators and Barriers to Physical Activity Questionnaire (FasBarPAQ)
Source: Rheumatol Int. 2022 Dec 7;43(2):303–14. doi: 10.1007/s00296-022-05252-8 (PMC9734883; doi:10.1007/s00296-022-05252-8)
Supplement: Supplementary file 2 — Supplementary file2 (PDF 171 KB) [file 296_2022_5252_MOESM2_ESM.pdf]

Videm V, Houge IS, Hoff M: Persons with rheumatoid arthritis have higher barriers to physical activity than controls – a cross-sectional study using the Facilitators and Barriers to Physical Activity Questionnaire (FasBarPAQ)
Rheumatology International
Corresponding author: Vibeke Videm, Department of Clinical and Molecular Medicine, NTNU – Norwegian University of Science and Technology and Department of Immunology and Transfusion Medicine, St. Olavs University Hospital, Trondheim, Norway. E-mail: vibeke.videm@ntnu.no

Online Resource 2: Mean FarBarPAQ item scores from blood donors (n=293)

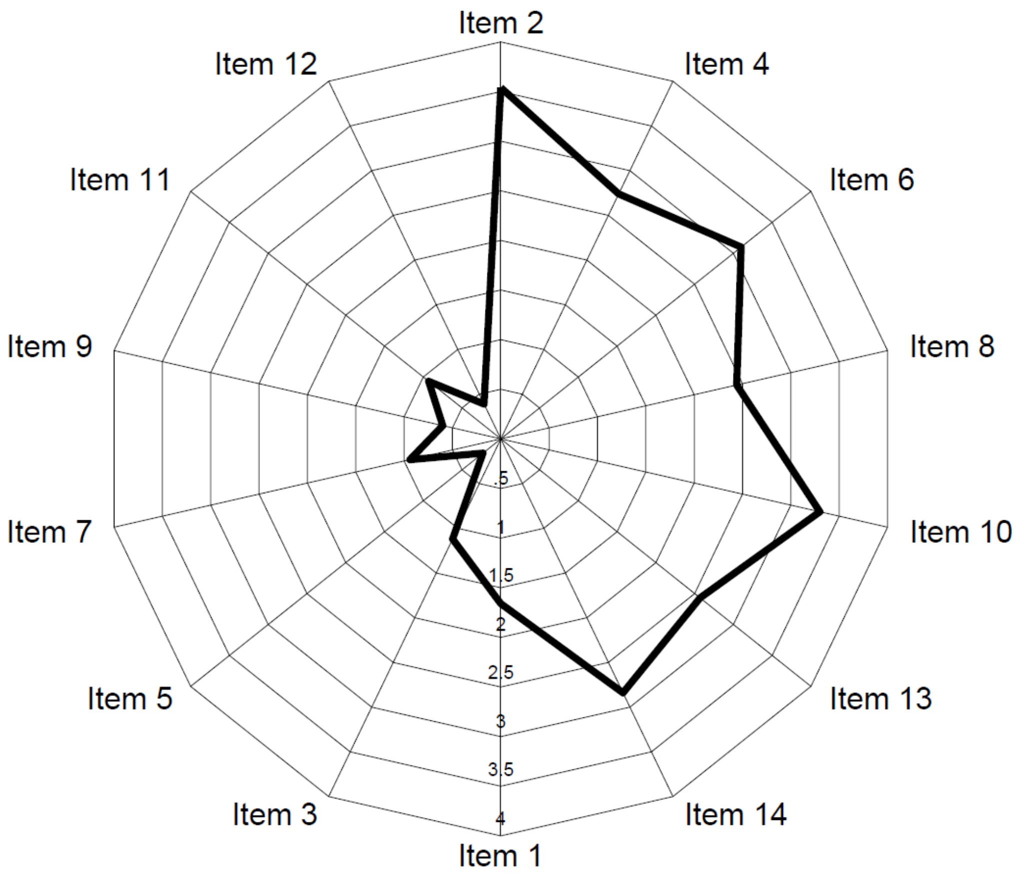

The black line indicates mean item scores, which may be used as a reference. Comparison with individual scores for persons with RA may help identify person-specific themes for intervention. Other reference populations may have other mean scores.

Items 2, 4, 6, 8, 10, 13, and 14 (right-hand side of diagram) represent facilitators.
Items 1, 3, 5, 7, 9, 11, and 12 (left-hand side of diagram) represent barriers.
