# Supplementary material for: Persons with rheumatoid arthritis have higher barriers to physical activity than controls: a cross-sectional study using the Facilitators and Barriers to Physical Activity Questionnaire (FasBarPAQ)
Source: Rheumatol Int. 2022 Dec 7;43(2):303–14. doi: 10.1007/s00296-022-05252-8 (PMC9734883; doi:10.1007/s00296-022-05252-8)
Supplement: Supplementary file 3 — Supplementary file3 (PDF 114 KB) [file 296_2022_5252_MOESM3_ESM.pdf]

Videm V, Houge IS, Hoff M: Persons with rheumatoid arthritis have higher barriers to physical activity than controls – a cross-sectional study using the Facilitators and Barriers to Physical Activity Questionnaire (FasBarPAQ)
Rheumatology International
Corresponding author: Vibeke Videm, Department of Clinical and Molecular Medicine, NTNU – Norwegian University of Science and Technology and Department of Immunology and Transfusion Medicine, St. Olavs University Hospital, Trondheim, Norway. E-mail: vibeke.videm@ntnu.no

Online Resource 3: Validation of the FasBarPAQ

Face validity – we are measuring what we intend to measure
The items for the FasBarPAQ were selected through several steps. In a pilot study, persons with rheumatoid arthritis (RA, n=93) were invited to give brief free-text input on factors that facilitate or act as barriers to their engagement in physical activity (PA). Based on common themes and an extensive literature search which also included publications regarding the general public and persons with other chronic diseases such as heart disease and cancer, the present 14 items were selected. In another pilot study, wording and completeness of replies were anonymously tested in university students (n=308). No problematic issues were identified.

Convergent validity – the scores correlate with other relevant variables
Based on the literature, we expected that facilitators and barriers to physical activity would be associated with psychological factors. For evaluation, depression and perceived stress were chosen. The present study showed associations in the predicted directions, especially for the barriers.

Divergent validity – the scores do not strongly correlate with each other
We expected that facilitators and barriers were two different concepts, albeit partly overlapping as people may evaluate the same aspect in opposite directions. For example, physical activity may be fun for some, but not at all for others. Likewise, many persons prefer social physical activities, whereas some find that the presence of others provokes negative concerns regarding their body shape or fitness level. The present study confirmed that the Facilitators and Barriers scores were weakly correlated ($R=-0.15$, $p<0.001$).

Predictive validity – the scores predict an important relevant outcome
We expected that facilitators and barriers to physical activity would impact on how much activity at different intensities a person performs. In turn, this should influence on whether they fulfill the current recommendations for physical activity or not. The present study confirmed that higher Facilitators scores and lower Barriers scores were significantly associated with fulfillment of the recommendations both in persons with RA and controls. The area under the receiver operating characteristic curve (AUC) was 0.77 (95% confidence interval: 0.73, 0.81), which indicates good discrimination. However, the FasBarPAQ is not designed to predict this outcome, so this analysis was performed as part of validation of the questionnaire.

Other performance measures
Many scores are developed using exploratory and confirmatory factor analysis. The aim is then to find a group of items that all represent a single common underlying concept, for example either facilitators or barriers to physical activity. Given that the same item could be a facilitator for some persons and a barrier for others and that people would show varying individual preferences, we

expected that factor analysis would not be suitable for the present questionnaire data. This was confirmed during the pilot phases of the FasBarPAQ development. To avoid problems and inconsistencies, we used the method presented in the Main paper to score the questionnaire responses. Traditional methods to evaluate factor analysis-based scores are therefore not relevant for the FasBarPAQ. This includes investigation of whether all items in a score load on the same factor, internal factor consistency (often measured with Cronbach's alpha), and factor structure stability among populations.

Because there is no "gold standard" to evaluate facilitators and barriers to physical activity, it is impossible to measure the performance of the FasBarPAQ with respect to sensitivity, specificity, or positive / negative predictive values.
